# Movement Patterns and Residency of the Critically Endangered Horseshoe Crab *Tachypleus tridentatus* in a Semi-Enclosed Bay Determined Using Acoustic Telemetry

**DOI:** 10.1371/journal.pone.0147429

**Published:** 2016-02-10

**Authors:** Toshifumi Wada, Takahiro Mitsushio, Shinya Inoue, Hiroko Koike, Ryo Kawabe

**Affiliations:** 1 Institute of Natural and Environmental Sciences, University of Hyogo, Sanda, Hyogo, Japan; 2 Fukuoka Prefectural Fisheries High School, Fukutsu, Fukuoka, Japan; 3 Kyushu Technological University, Kitakyushu, Fukuoka, Japan; 4 Graduate School of Social and Cultural Studies, Kyushu University, Fukuoka, Japan; 5 Institute for East China Sea Research, Graduate School of Fisheries and Environmental Sciences, Nagasaki University, Nagasaki, Japan; Australian Institute of Marine Science, AUSTRALIA

## Abstract

The horseshoe crab *Tachypleus tridentatus* is critically endangered in Japan due to rapidly decreasing numbers resulting from the loss of tidal flats and sandy beaches, and the deterioration of coastal environments. We monitored the year-round migratory patterns and residency of this species in a coastal embayment at Tsuyazaki, Japan, using acoustic telemetry. Total 20 adult crabs (15 males and 5 females) were tagged with ultrasonic transmitters and tracked during two periods (2006–2008; *n* = 10 and 2007–2009; *n* = 10). Adult crabs were more active during periods of higher water temperatures and their activity peaked in July, during the spawning period. Water temperature appeared to be one of the key factors influencing the movement patterns for the species. Moreover, the crabs tended to be more active at night than in the day. The nocturnal activity pattern was clearly evident before and during the reproductive period (May–August). Tracking data also showed that one pair-bond was maintained for a maximum of 17 days after the pair-bonded female had spawned. Overall, 11 males (73% of 15 individuals) remained in the bay area over winter, whereas three females (60% of 5 individuals) overwintered outside of the bay. Telemetry data showed that over 60% (13 of 20) of tagged crabs overwintered within the bay where there are sandy beaches, mudflats, and scattered seagrass beds. This year-round residence by adult *T*. *tridentatus* in the bay area identifies it as a critical habitat for the management of this species, regardless of life-stage. Not only is it a comprehensive management strategy that effectively reflects this species’ habitat use patterns but also its implementation, such as the establishment of a protected area, would contribute to its conservation.

## Introduction

The horseshoe crab *Tachypleus tridentatus* is distributed throughout coastal waters from Southeast Asia to the western part of Japan [1]. This species has been decreasing in numbers because of the loss of tidal flats and sandy beaches, and the deterioration of coastal environments in Japan, Taiwan, and Hong Kong [2–4]. The Japanese population is included on the Red List and in the Red Data Book of Japan as critically endangered (CR + EN) [5, 6]. Moreover, there are some genetic subdivisions among the Japanese populations [7].

*Tachypleus tridentatus* requires different coastal habitats over its life cycle (reviewed in [1]). Specifically, reproductive pairs breed on sandy beaches at high tide from July to August [8, 9]. Hatchings leave the beach areas and move to the adjacent mudflats [10, 11]. Juveniles (small instars) develop on the mudflats until they reach a carapace width of approximately 7 cm, with molting every year [12, 13]. Although the subsequent movement and later habitat use by subadults are still unknown, they are presumed to inhabit seagrass beds (*Zostera* spp.) and foreshore areas [1, 14]. During several months including the spawning period, adult and juvenile crabs are often easily observed in the shallows and intertidal areas. However, an understanding of their movement patterns and habitat use during the remainder of the year is largely lacking because of the difficulty in defining fine-scale and long-term movement patterns of individuals underwater.

Recently, acoustic telemetry using automated monitoring receivers and coded ultrasonic transmitters has been applied to studying the movements and habitat use of various aquatic animals including teleosts, chondrichthians, crustaceans and cephalopods (reviewed in [15]). The use of acoustic tracking systems has provided valuable insight into migration patterns and habitat use. In the American horseshoe crab *Limulus polyphemus*, some recent investigations using acoustic telemetry systems have examined seasonal movements and overwintering sites in natural habitats [16–20]. James-Pirri [18] and Schaller *et al*. [19] showed that the crabs along the northeast coast of the United States moved up into estuaries in spring when water temperatures exceeded 11°C; after spawning some moved back down the estuaries and overwintered in deeper bay waters. Knowledge of year-round habitats of wild animals has provided the clues and background essential to their conservation and management. Previous acoustic tracking surveys of the critically endangered *T*. *tridentatus* have noted that adult crabs move within Moriye Bay at Oita prefecture, Japan, after spawning [21, 22]. However, the movement patterns and habitat use of this species throughout the year have not been explicitly addressed.

In the present study, we investigated the year-round movement patterns of *T*. *tridentatus* around a coastal embayment in Japan using a modern acoustic telemetry system. The aims of this study were to identify the movement patterns of crabs and their habitat use, including their overwintering grounds, around a coastal embayment.

## Materials and Methods

### Study site and experimental animals

**Fig 1:**
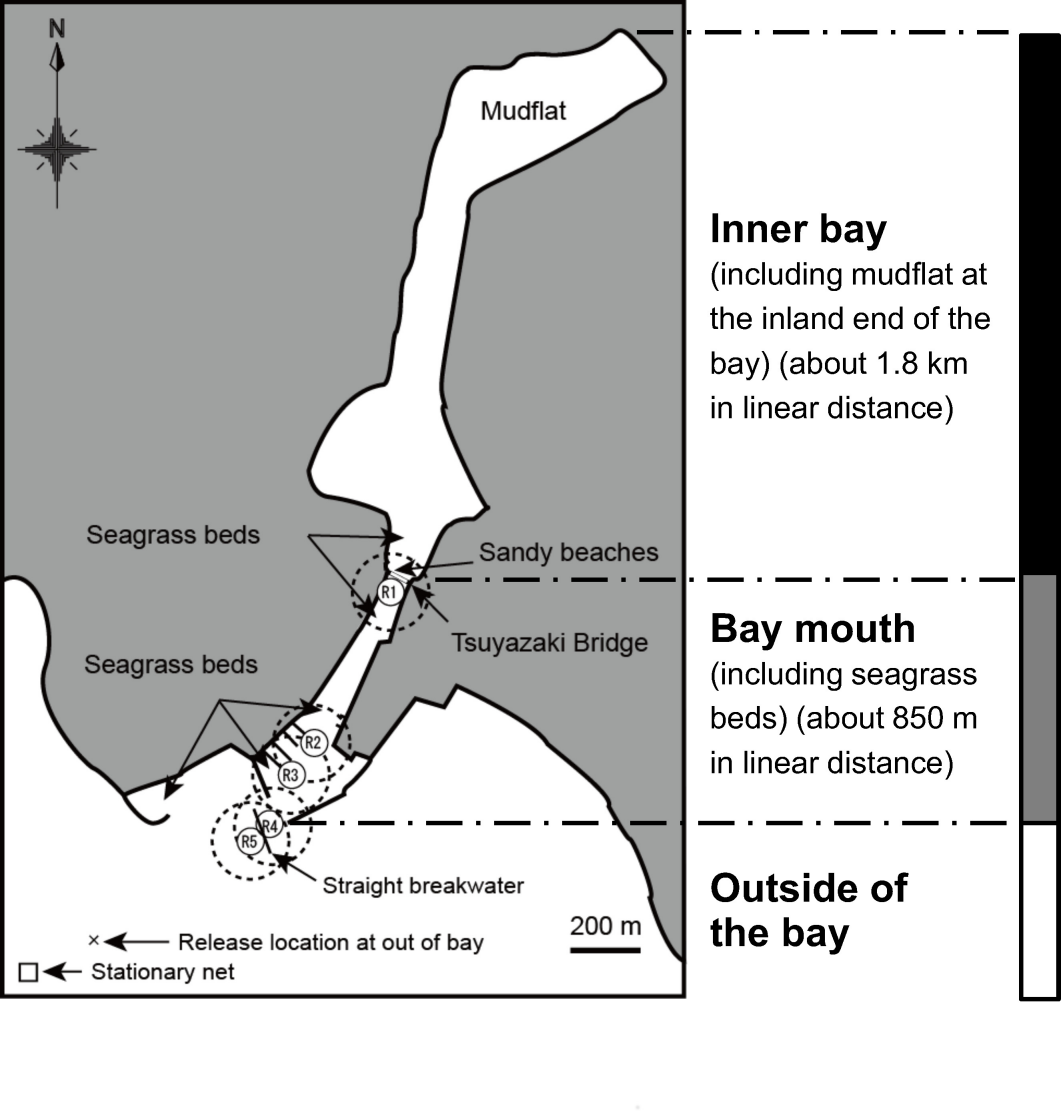
Map showing the locations of the Tsuyazaki Bay study area (33°47′N, 130°27′E; further map data are available in the OpenStreetMap.org website), locations of acoustic receivers (R1–R5), and the three parts of the bay area delineated for this study. Dashed circles represent the expected detection ranges for the coded ultrasonic transmitters.

This study was conducted around Tsuyazaki Bay in Fukuoka prefecture, Kyushu Island, which is in western Japan (Fig 1). All fieldwork was carried out in compliance with local laws. The project had the full support of the local government (Fukutsu City) and its citizens [9, 13] because the study area was not on record as one of the important habitats and spawning sites for the critically endangered horseshoe crab *T*. *tridentatus*. Because of the lack of information about the species, this habitat area was not protected and did not require field permits for access.

The semi-enclosed Tsuyazaki Bay is approximately 3 km in length and tens to several hundred meters in width (maximum, 550 m). A straight breakwater (about 150 m long) has been built near the bay mouth. The majority of the area of the bay is composed of intertidal sandy beaches, some scattered seagrass beds (mostly *Zostera japonica*) and tidal mudflats. The sandy beaches near Tsuyazaki Bridge and the mudflat at the inland end of the bay are used by *T*. *tridentatus* as spawning and nursery grounds, respectively [9, 13]. For this study, three habitats were identified, largely on the basis of their different environments: the inner bay including tidal mudflats, the bay mouth where there are some scattered seagrass beds, and outside the bay (Fig 1). The boundaries between these habitats were delineated by the locations of Tsuyazaki Bridge and the straight breakwater.

In order to follow year-round movement patterns, 20 adult crabs (15 males, carapace width [mean ± SD]: 23.6 ± 1.4 cm; 5 females: 29.6 ± 1.4 cm) were tagged with ultrasonic transmitters (Table 1). All experiments were conducted in accordance with the Japan Ethological Society guidelines for the experimental use of animals (http://www.ethology.jp/guideline.pdf). The crabs were caught by a small stationary net outside of the bay (up to 1 km offshore from the bay mouth; Fig 1) or by hand at the sandy spawning beaches within the bay, during April–July 2006 and April–August 2007. Five of the crabs (tag IDs 188–192) caught by stationary net were kept in a net cage (diameter, 2 m) until tagging; the cage was submerged on the bay bottom for a few weeks at most. The sex of tagged crabs was determined by observations of the prosoma and pedipalps. To avoid losing the transmitters during tagging experiments, only adult specimens that had completed the final molt were selected, because annual molting ends with maturation [1].

**Table 1:** Details of tagged horseshoe crabs *Tachypleus tridentatus*.

ID no.	Sex	Carapace width (cm)	Pair or unpair	Tagging and release date	Release location	Last detected date
188	Male	25.2	unpair	18-Jun-2006	out of bay	12-Jul-2006
189	Male	26.9	unpair	18-Jun-2006	out of bay	5-Aug-2006
190	Male	24.6	unpair	25-Jun-2006	out of bay	16-Jul-2006
191	Male	22.9	unpair	5-Jul-2006	out of bay	9-Oct-2006
192	Male	20.8	unpair	5-Jul-2006	out of bay	3-Feb-2008
193	Male	24.4	pair	9-Jul-2006	bay area	31-May-2007
194	Male	23.5	pair	9-Jul-2006	bay area	10-Jul-2006
195	Male	24.0	unpair	9-Jul-2006	bay area	11-Jul-2007
196	Male	23.8	pair	10-Jul-2006	bay area	19-Jun-2007
187	Male	24.6	pair	10-Jul-2006	bay area	9-Jun-2008
4909	Female	31.4	pair with ID4910	29-Jun-2007	bay area	15-Jul-2008
4910	Male	23.3	pair with ID4909	29-Jun-2007	bay area	23-Jul-2007
4911	Female	29.8	pair with ID4912	29-Jun-2007	bay area	1-Sep-2007
4912	Male	21.4	pair with ID4911	29-Jun-2007	bay area	13-Oct-2007
4913	Female	30.0	pair with ID4914	12-Jul-2007	bay area	18-Jul-2007
4914	Male	23.1	pair with ID4913	12-Jul-2007	bay area	12-May-2008
4915	Female	27.2	pair with ID4916	1-Aug-2007	bay area	30-Sep-2007
4916	Male	23.0	pair with ID4915	1-Aug-2007	bay area	6-Dec-2009
4917	Female	29.7	unpair	10-Aug-2007	bay area	6-Dec-2009
4918	Male	22.8	unpair	10-Aug-2007	bay area	6-Dec-2009

### Telemetry system and tagging with acoustic transmitters

Transmitter models V13 (13-mm diameter, 33-mm length, 6 g in water) and V16 (16-mm diameter, 68-mm length, 10 g in water) (Vemco Ltd, Nova Scotia, Canada) were used for tracking animals during 2006–2008 and 2007–2009, respectively. These tags transmit a unique coded pulse at a frequency of 69 kHz at random intervals every 10–30 s, with predicted lifetimes of 289 days (V13) or 1542 days (V16). The protocol used was almost the same as that used for acoustic tagging of the American horseshoe crab *L*. *polyphemus* by Brousseau *et al*. [16]. Briefly, crabs were gently placed in a plastic container (50 × 120 × 20 cm; S1 Fig) with seawater for tagging and then the dorsal carapace was exposed. The carapace width was measured to within 0.1 cm and an acoustic transmitter was attached to the extracardiac region of the dorsal carapace. The attachment position was first wiped clean and dried. The transmitter was attached to the dorsal carapace with silicone adhesive (silicone sealant 8060; Cemedine Corp., Tokyo, Japan). After the transmitter was attached, the adhesive was allowed to cure sufficiently before submerging the tagged crabs, still in the container, under seawater, where they were kept for more than 1 h prior to release. This tagging procedure, including the curing time for the silicone adhesive, took about 1.5 h for each crab. During visual observation after the tagging, there were no behavioral changes or problems otherwise for the crab’s swimming and digging. All tagged crabs were released in the same locations where they were captured. In 2007–2009, four males and four females were tagged as attached male–female pairs (S2 Fig), although one pair separated during the tag-attachment procedure. Details of the tagging and release are shown in Table 1.

Tagged crabs were tracked using continuously monitoring receivers (VR2; Vemco), which are single-channel, omni-directional units that recorded the time, date, and identity of tags within their detection range [15]. Before the experiments of 2006–2008, four receivers (R1–R4) were deployed along a route from the inner bay to the bay mouth; one additional receiver (R5) was placed outside of the bay before the observations in 2007–2009 (Fig 1). Each receiver was fastened to PVC pipe by plastic banding; the pipe was attached to a concrete block (33 × 39 × 17 cm) as a mooring anchor and submerged at heights of approximately 1 m above the bay bottom to prevent becoming buried in the bottom sediment. The receivers were placed near a marina or by a floating landmark.

The location of each listening station was determined on the basis of the detection experiments (Fig 1). The decisions about detector spacing were based on a desire to monitor when tagged crabs entered and left the bay. Before the tagging survey, the tag detection range in the bay was found to be up to 150 m for both V13 and V16 transmitters. In order to determine the location of tagged crabs from the number of times they were detected by each receiver, three receivers were deployed at the bay mouth with overlap of their expected detection range. Moreover, to ascertain whether the acoustic signals would be received sequentially at each receiver with the passage of a crab toward the inner bay from outside of the bay under suboptimal conditions such as during rainfall or at low tide, a transmitter was repeatedly towed by boat to resemble the movement of a crab, after the listening stations had been deployed.

All receivers continuously monitored the passage of tagged individuals until 19 December 2009. The data from each receiver were downloaded at least once every 3 months during the first years (2006–2008) and thereafter once every 6 months. Water temperature was recorded using two temperature loggers (Tidbit v2; Onset Corp., Bourne, Massachusetts, USA), which were deployed with the receivers in the inner bay (R1) and outside the bay (R5) over a year beginning in September 2008. For this study, the water temperature data from the sensor outside of the bay were used because some of the data from the inner bay could not be downloaded because of a mechanical failure. The difference in water temperature between the inner bay and outside of the bay averaged less than 1°C during the first two months before the mechanical failure.

### Data analysis

According to Heupel *et al*. [15], continuous data obtained from an acoustic array with overlapping detection ranges provide some indication of direction of movement based on the sequence of detections. Three receivers were deployed (R2, R3, R4) with overlapping detection ranges to monitor the direction of crab movement and their residency in the bay mouth (Fig 1). Receiver R5 was used to monitor crabs outside of the bay only because the acoustic signals were blocked by the breakwater. To determine site fidelity of tagged crabs, receivers R1 and R4 were deployed at the boundaries between the three habitats. When sequential detections by receivers in the bay mouth were interrupted for more than 1 h at the boundaries between habitats (detectors R1 and R4), this was defined as a crab movement between the habitats for the purposes of this study. The crab movements were classified as movements from the inner bay to the bay mouth or from the bay mouth to outside of the bay. In this study, because data from some V13 transmitters were received after the predicted lifetime of the transmitters, these sequence detection data were included with the study results.

To examine seasonal changes in habitat preference, four time periods were used to designate the seasons [18, 19]: winter, December to March; spring, April to June; summer, July to August; and fall, September to November. Day and night were defined as 0800–1800 and 2000–0600 h local time, respectively. To examine annual and diel movement patterns of horseshoe crabs, the number of movements of each crab between the habitats was compared during each season and between day and night, using the Friedman nonparametric test and the Mann–Whitney *U*-test, respectively.

To investigate the residency of tagged crabs in the three habitats (inner bay, bay mouth, and outside the bay; Fig 1), the occupancy in each habitat during the monitoring period of the study was calculated on the basis of the site fidelity of individuals. The time animals spent within each area was calculated using data for individual crabs detected by each receiver [23]. The percentage occupancy (*R*, %) was estimated as follows:

$$R=N_{1}/N_{2}\times 100$$

where *N*_1_ is the time spent within each area for a tagged crab and *N*_2_ is the monitoring period based on the expected battery life of the transmitter. The detection data within a year from some V13 transmitters received sequentially after the predicted lifetime of the transmitters were included in this analysis. In accordance with the above-mentioned definition of crab movement between the habitats, the residence time (h) was calculated for which a tagged crab stayed within each habitat. To confirm the presence of tagged crabs in areas outside of the detection area (i.e. the area between R1 and R2, and the east side of the bay mouth) after all receivers lost touch with a transmitter, acoustic monitoring surveys were conducted by submerging another VR2 receiver for a few hours at various locations in these areas at least once every 6 months during this study period.

The residence times for each tagged crab were summed for each season. The summed residence times for tagged crabs were compared using the Kruskal–Wallis test to identify seasonal differences in their habitat use. For this analysis, only data from the first year after tagging were used as the monitoring period for the 2007–2009 experiment, because some tagged crabs continued to be detected at almost the same locations starting in the second year, including during the usual period of activity. The overwintering area for each crab was estimated by its location at the beginning of each November, because animal movement lasted only until late October (see Results). Statistical analyses were performed using the statistical software package SPSS 20.0 (IBM Corp., NY, USA); a *P*-value less than 0.05 indicated statistical significance.

## Results

### General results

**Fig 2:**
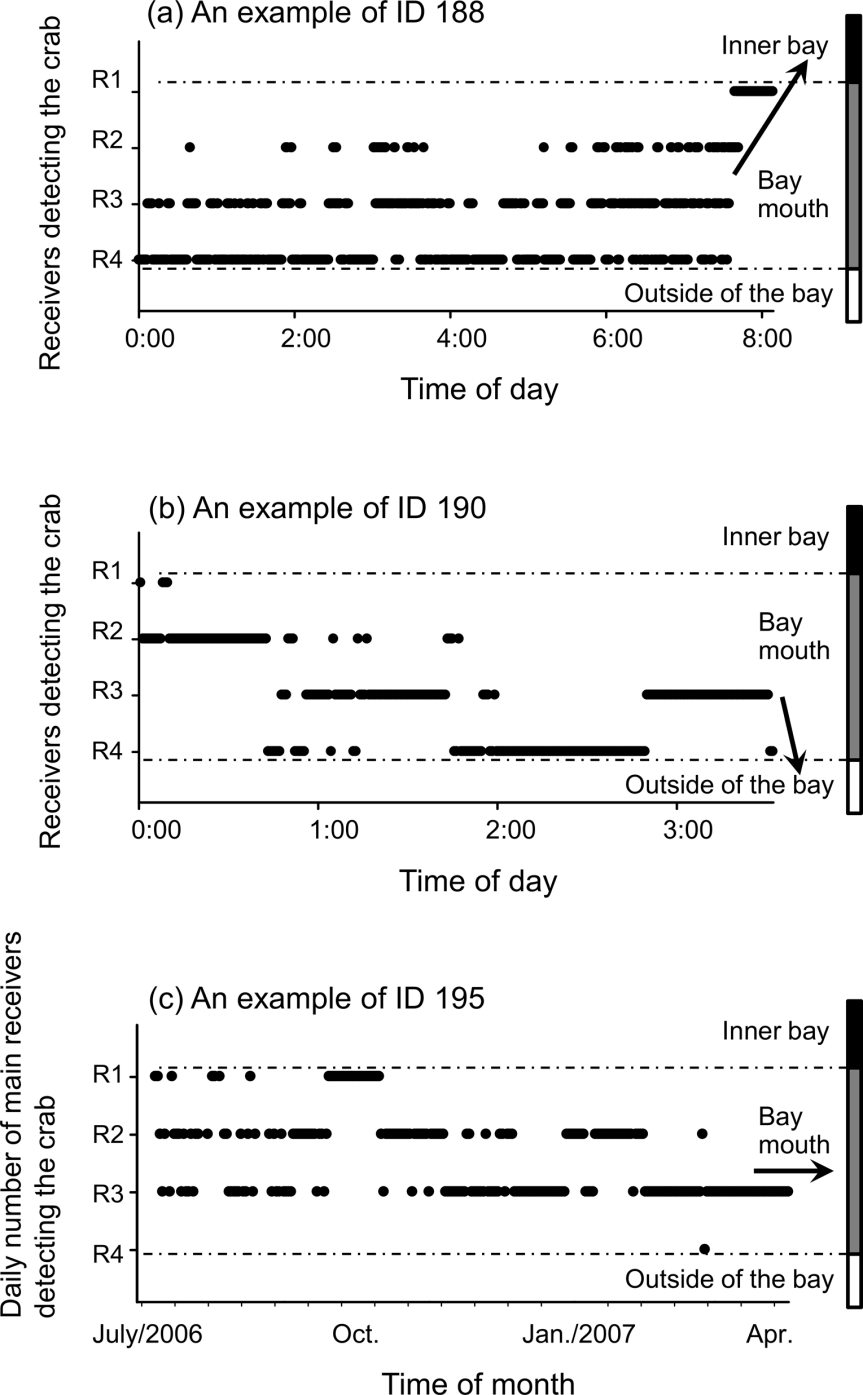
Examples of acoustic telemetry data reflecting behavioral patterns of the horseshoe crab *Tachypleus tridentatus*. Each filled circle indicates the time and receiver detecting a tagged crab. (a), (b) Examples of crab movement to the inner bay (focusing on 12 July for tag ID 188) or out of the bay (16 July for ID 190), respectively. Time series data show detections by multiple receivers over a portion of a day. The arrows represent the direction of crab movement between habitats. (c) An example of crab residence in the bay mouth (ID 195), with daily time series data showing the main receiver with hourly detections of a crab. The arrow represents the prolonged residence in the bay mouth.

Each of the 10 crabs tagged during 2006–2008 and 2007–2009 were monitored for 2–700 days (mean, 253.6 days) or 6–859 days (mean, 351.1 days), respectively. A summary of the acoustic tagging survey data is presented in Tables 1 and 2. During 2006–2008, five tagged crabs (tag IDs 188–192) released outside the bay moved into the bay mouth area within 4 days after release. Four of these five tagged crabs subsequently moved into the inner bay (IDs 188, 189, 191) (Fig 2A) or stayed within the bay mouth (ID 192), and the fifth (ID 190) went outside of the bay after about 20 days (Fig 2B). During 2006–2008, four of five tagged crabs (IDs 187, 194, 195, 196) released inside the bay, stayed between the bay mouth and the inner bay during the whole monitoring period (Fig 2C). One tagged crab (ID 193) moved outside of the bay about 8 days after release and returned to the bay in spring of the following year.

**Table 2:** The number of movements between three bay areas, residency, and estimated overwintering areas of tagged horseshoe crabs *Tachypleus tridentatus* in Tsuyazaki, Japan.

ID no.	No. of migrations		Residency (%)			Estimated overwintering area		
	Inner bay to bay mouth	Bay mouth to out of bay	Inner bay	Bay mouth	Out of bay	2006	2007	2008
188	5	11	92.6	3.3	4.1	Inner bay	-	-
189	1	3	90.8	1.6	7.6	Inner bay	-	-
190	8	2	1.2	5.8	93.0	Out of bay	-	-
191	1	5	71.9	25.2	2.9	Inner bay	-	-
192	1	1	0	100	<0.1	Bay mouth	-	-
193	3	11	1.7	0.8	97.5	Out of bay	-	-
194	0	0	100	0	0	Inner bay	-	-
195	39	10	12.3	83.6	4.1	Bay mouth	-	-
196	1	0	80.0	20.0	0	Inner bay	-	-
187	2	6	1.6	94.5	3.9	Bay mouth	-	-
4909	5	13	0.1	1.3	98.6	-	Out of bay	Out of bay
4910	1	1	0.5	<0.1	99.5	-	Out of bay	-
4911	19	0	91.2	8.8	0	-	Inner bay	-
4912	9	9	1.5	22.6	75.9	-	Out of bay	-
4913	3	1	1.2	0.3	98.5	-	Out of bay	-
4914	13	14	24.1	74.0	1.9	-	Bay mouth	Inner bay
4915	3	15	4.2	11.5	84.3	-	Out of bay	-
4916	5	0	11.5	88.5	0	-	Bay mouth	-
4917	0	0	100	0	0	-	Inner bay	-
4918	8	0	99.7	0.3	0	-	Inner bay	-

(-) = no data

**Fig 3:**
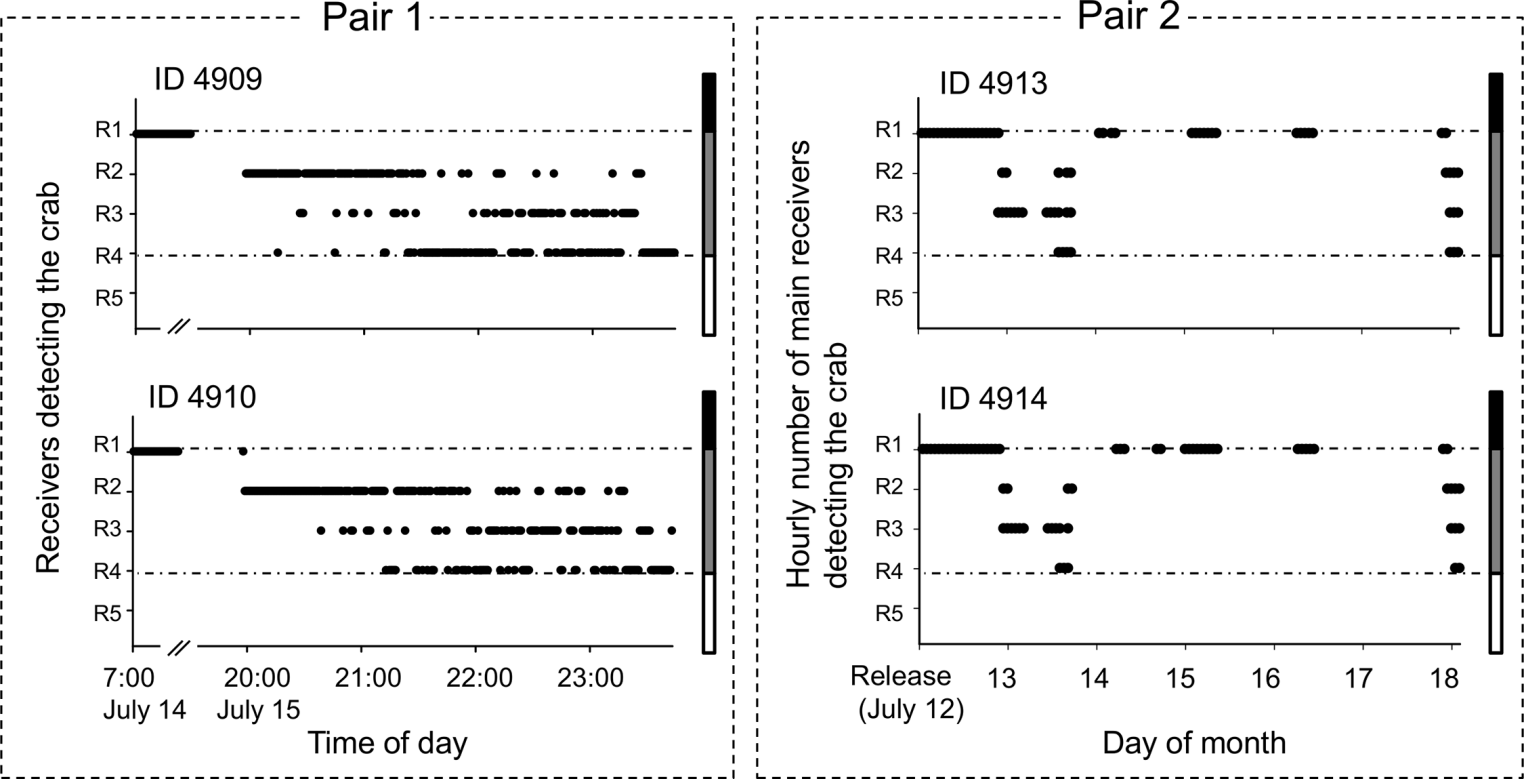
Time series showing the number of the receivers detecting pairs of *Tachypleus tridentatus*.

During 2007–2009, 10 tagged crabs were released inside the bay. Two of the four tagged pairs (IDs 4909 and 4910, and IDs 4913 and 4914), which were released while the male was still attached to the female (S2 Fig), appeared to move together until about 17 and 6 days after release, respectively, because each member of the pairs was detected by the same listening station at almost the same time (Fig 3). The other two pairs (IDs 4911 and 4912, and IDs 4915 and 4916) seemed to separate approximately 7 and 10 days after release, respectively, because at that time the members of each pair were detected by different listening stations almost simultaneously, or one member of the pair was no longer detected. During 2007–2009, four females (IDs 4909, 4912, 4913, 4915) and two males (IDs 4910 and 4914) went outside of the bay after release; crabs 4909 and 4914 returned to the bay mouth in June of the following year and in September 2007, respectively. The other four tagged crabs were continuously detected in the bay mouth during the monitoring period or disappeared after detection at receiver R1 returning to the inner bay.

### Migratory patterns and residency around the bay

**Fig 4:**
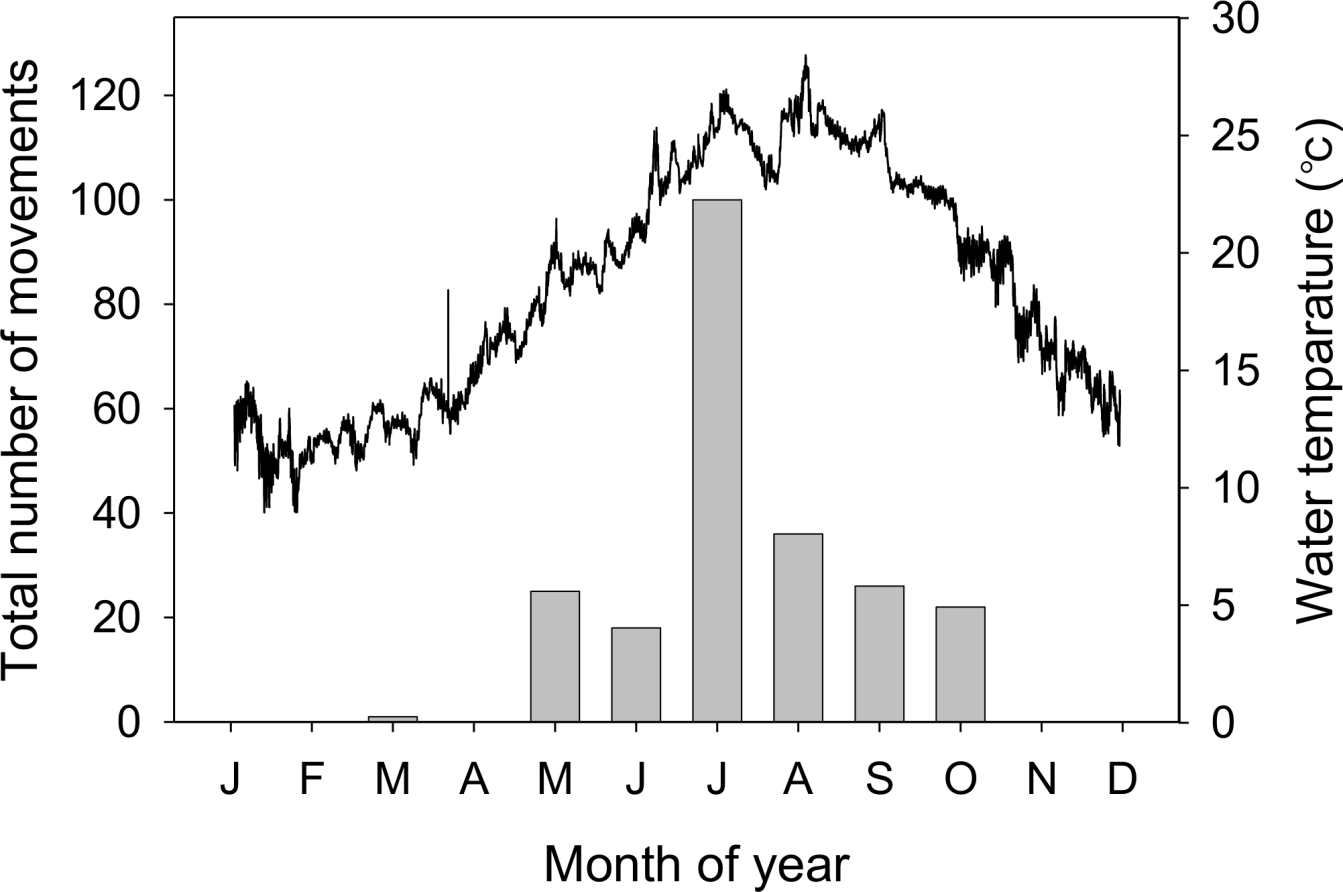
Monthly total number of movements between habitats of tagged *Tachypleus tridentatus* summed over the study period. Solid black line shows the hourly water temperature in 2008–2009 at the location of receiver R5, just outside Tsuyazaki Bay.

**Fig 5:**
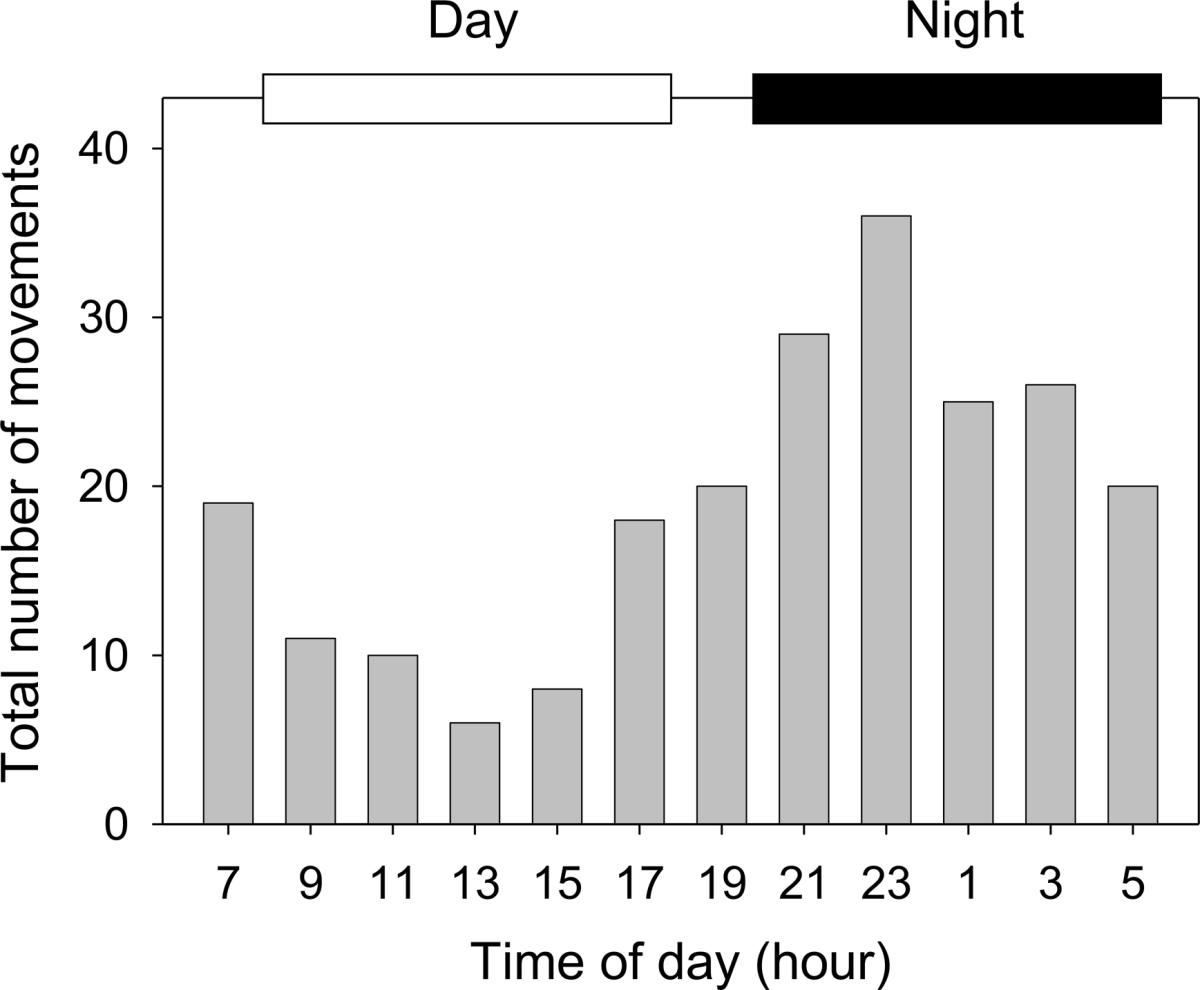
Hourly total number of movements between habitats of tagged *Tachypleus tridentatus* summed over the study period. White and black bars represent the day (0800–1800) and night (2000–0600) periods, respectively.

In total, 18 tagged crabs moved into or out of the bay area (inner bay to bay mouth [mean times per crab ± SD]: 7.0 ± 9.4 times, range: 0–39 times; bay mouth to outside of the bay: 5.7 ± 5.5 times, range: 0–15 times). Almost all movements occurred between 4 May and 25 October (Fig 4), except for one datum recorded in March. The water temperature around Tsuyazaki Bay (range, 16.8–28.4°C) increased to 20°C in early May when the crabs began to move between habitats, and fell below 20°C in late October at the end of the period of crab movements (Fig 4). The tagged crabs (*n* = 18) moved more frequently in summer than in other seasons (spring: mean times per crab ± SD, 2.39 ± 4.39 times; summer: 7.56 ± 6.87 times; fall: 2.67 ± 6.19 times) (ANOVA repeated measures: *P* < 0.01; Friedman’s test: χ^2^ = 17.5, *P* < 0.01). More than 60% of total movements (*n* = 229) between habitats were recorded in summer (July and August). The number of movements significantly increased during night (2000–0600: mean times per crab ± SD, 7.6 ± 7.2 times, range: 0–30 times) compared to day (0800–1800: 2.9 ± 3.5 times, 0–12 times) (Mann–Whitney *U* test, *Z* = –2.49, *P* < 0.05; Fig 5). There was a significant pattern of increased nocturnal movement during the summer and spring (Mann–Whitney *U* test, *Z* = –2.68 and –2.17, *P* < 0.01 and *P* < 0.05, respectively), but the trend was not evident during the fall (*Z* = –1.12, *P* > 0.05).

**Fig 6:**
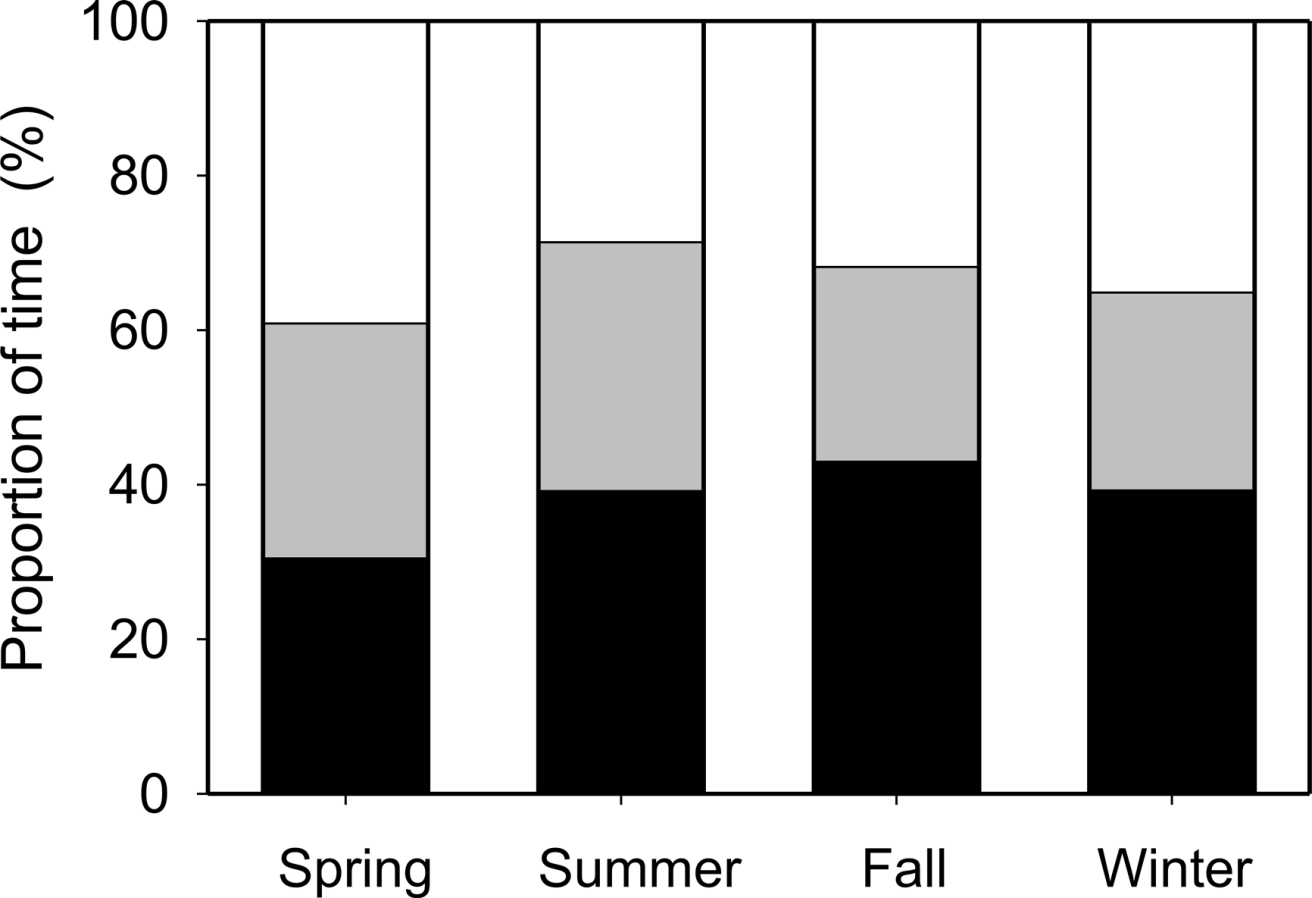
Seasonal variation in the mean proportion of time spent in each of three bay areas calculated from the residence time of individual tagged crabs *Tachypleus tridentatus*. Inner bay, black bars; bay mouth, grey bars; outside of the bay, white bars.

Thirteen of twenty tagged crabs (65%) spent the majority of their time within Tsuyazaki Bay habitats (bay mouth or inner bay) during the survey period (Table 2). Although the mean proportion of seasonal utilization for each area ranged from 25.6% to 39.0% (25.6–38.1% in inner bay, 30.2–36.3% in bay mouth, and 28.5–39.0% outside of the bay), there was no significant difference in seasonal residency between the three habitats (Kruskal–Wallis test, all *P* > 0.05; Fig 6).

Thirteen of the twenty tagged crabs overwintered in Tsuyazaki Bay (bay mouth or inner bay) (Table 2). Five of these thirteen individuals were continuously detected by the three receivers at the bay mouth (R2, R3, R4), even during winter. The last detections of the other nine crabs before winter were recorded at receiver R1 at the entrance to the inner bay (including one male that was wintering in both the bay mouth and inner bay). Overall, 11 males (73% of 15 individuals) remained in the bay area over winter, whereas three females (60% of 5 individuals) overwintered outside of the bay.

## Discussion

Acoustic telemetry data made it possible to monitor the locations and movements of adult horseshoe crabs *T*. *tridentatus* in a coastal embayment; the data demonstrated that the majority of crabs inhabited the bay throughout the year. A previous study reported that the crabs used deeper offshore habitats (>20m depth) during winter [1], but little is known about their migration and habitat use except for along the shoreline during the reproductive period (June–August) [1, 8, 9]. Understanding the movement and habitat use patterns of threatened species is essential to effective conservation and management such as the establishment of a protected area.

Our tracking data provide evidence that over 60% of tagged crabs overwintered in the bay where there are sandy beaches, mudflats, and seagrass beds (Table 2). In fact, adult *T*. *tridentatus* were often found covered with mud on the intertidal mudflats or subtidal seabed during the winter and subsequent early spring (for example, S3 Fig). Previous studies have also shown that mudflats and seagrass beds are important maturation grounds for juvenile and subadult *T*. *tridentatus* [1, 3, 14, 24]. In addition to the importance of diverse coastal habitats as nurseries, the year-round residence by adult horseshoe crabs in the bay area identifies it as a critical habitat for the management of this species, regardless of its life-stage. Our results suggest the need for a comprehensive approach to conservation and spatial management reflecting the habitat utilization for the species overall, or for local populations.

Eighteen crabs tracked in this study provide additional insights into their movement patterns in the bay area. Crab movements began in May and lasted until late October, in the post-spawning period, and there was more activity during the period of higher water temperatures (Fig 3). This trend is consistent with other studies for the American horseshoe crab *L*. *polyphemus*, which begins to move into estuaries in spring when water temperatures exceed 10–11°C; the activity decreases in autumn [18, 19]. Thus, water temperature appears to be one of the key factors influencing the movement patterns for both of these horseshoe crab species.

Some previous studies of *T*. *tridentatus* have shown that the spawning activity peaks around the July new and full moons, with a semilunar rhythm [1, 8]. Other studies of *L*. *polyphemus* reported that their patterns of movement were an expression of the circadian rhythm of their visual sensitivity [25, 26]. In some recent studies, movement in *L*. *polyphemus* was closely related to the tidal cycle, light intensity, and water depth [27–31]. The present study shows that tagged crabs moved frequently before, during, and after the reproductive period (June–August), but any external rhythms or environmental cues inducing such migratory behavior were unresolved because short-range crab movements could not be detected at the bay mouth or in the inner bay.

Our results also indicate that the crabs tend to be more active at night than during the day (Fig 5). Such nocturnal activity patterns have been reported for mammals and reptiles and are often accounted for as being times for the animals to feed efficiently (e.g. [32, 33]). For the horseshoe crabs, which have a strong carapace and feed on benthos, it seems unlikely that they adopt a nocturnal pattern for foraging and to avoid the risk of predation. One plausible advantage of a nocturnal activity pattern in horseshoe crabs would be the avoidance of egg predation (as seen, for example, in sea turtles [34, 35]). The hypothesis of egg predation avoidance gains possible support from the absence of a nocturnal activity pattern during the post-spawning period (September–October).

The present study provides some findings on the pair-bonding of this species. Many monogamous animals such as birds and mammals have been known to maintain long-lasting pair-bonds [36, 37]. Social monogamy or pair living often involves a high cost of finding a new mate and highly competitive societies. Under these circumstances, prolonged pair bonds increase lifetime reproductive success [38]. Although the mating system of *T*. *tridentatus* is still unknown, our tracking data show that one pair-bond was maintained for a maximum of 17 days after the pair-bonded female had spawned (Fig 3). The male in a pair tightly grasps the opisthosoma of the female using modified prosomatic appendages, which implies that the disruption of the pair-bond depends on the male. Behavior towards potential new partners has been shown to differ between the sexes in a monogamous cichlid [39]. In the present study, more tagged males (73% of 15 individuals) than females (40% of 5 individuals) remained in the bay area over the winter. This sedentary tendency in males may result in the disruption of pair bonding. In contrast, the post-spawning females would show a preference for foraging over a wide range of coastal areas presumably to restore their depleted resources [18]. Future studies for *T*. *tridentatus* should address shifts in habitat use with special attention to sex differentiation.

The horseshoe crab *T*. *tridentatus* is thought to be sensitive to environmental changes resulting from human presence and activity [2–4]. As noted in a previous study [9], the decreasing number of reproductive pairs of the Tsuyazaki population visiting this study area is probably due to recent coastal developments such as the construction of a yacht harbor and breakwater. Identifying areas that are highly frequented by this species is a key priority for its conservation [40, 41]. Our study area is an important year-round habitat for *T*. *tridentatus* covering diverse coastal environments, but there is spatial overlap between human use and crab habitat. Future studies should confirm and define the predictable anthropogenic influence with a greater number of tagged crabs.

## Supporting Information

S1 Fig A pair of *Tachypleus tridentatus* in a plastic container, each tagged with an acoustic transmitter. (TIF)

S2 Fig A released pair of tagged *Tachypleus tridentatus*, each with an attached transmitter. (TIF)

S3 Fig A pair of *Tachypleus tridentatus* found covered with mud on the intertidal mudflats. (TIF)
