# Supplementary figures and images for: Movement Patterns and Residency of the Critically Endangered Horseshoe Crab Tachypleus tridentatus in a Semi-Enclosed Bay Determined Using Acoustic Telemetry
Source: PLoS One. 2016 Feb 10;11(2):e0147429. doi: 10.1371/journal.pone.0147429 (PMC4749169; doi:10.1371/journal.pone.0147429)

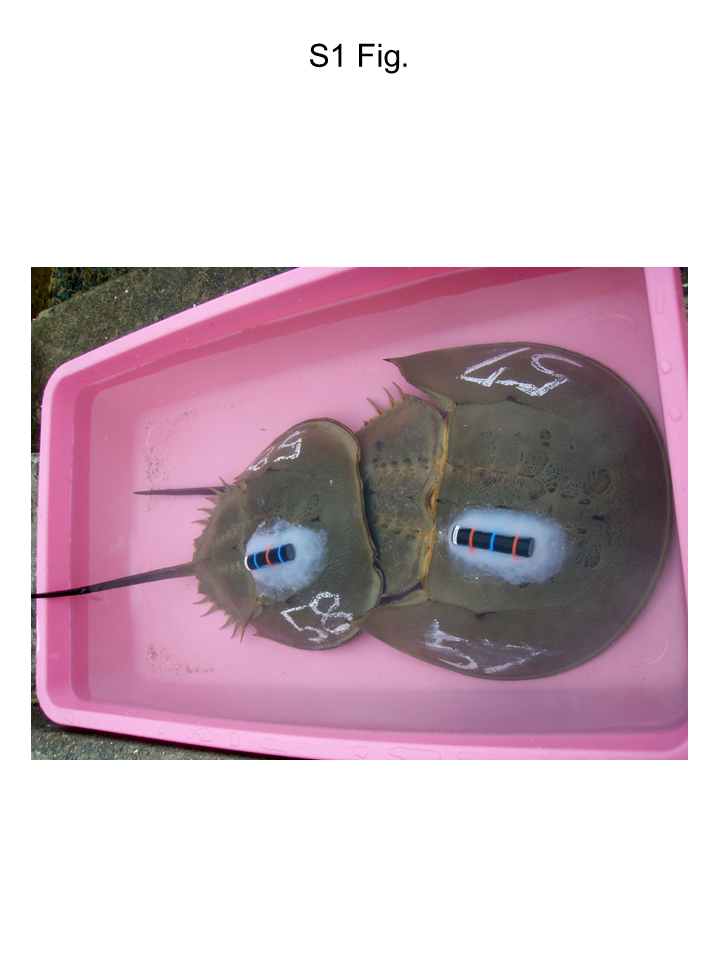

Supplement: S1 Fig — (TIF) [file pone.0147429.s001.tif]

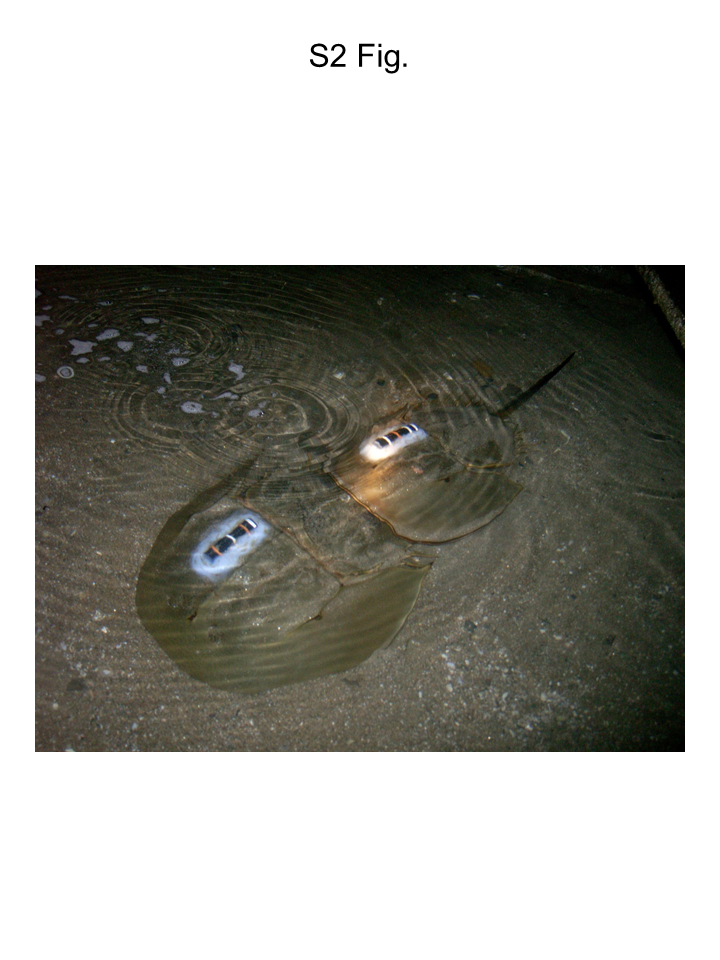

Supplement: S2 Fig — (TIF) [file pone.0147429.s002.tif]

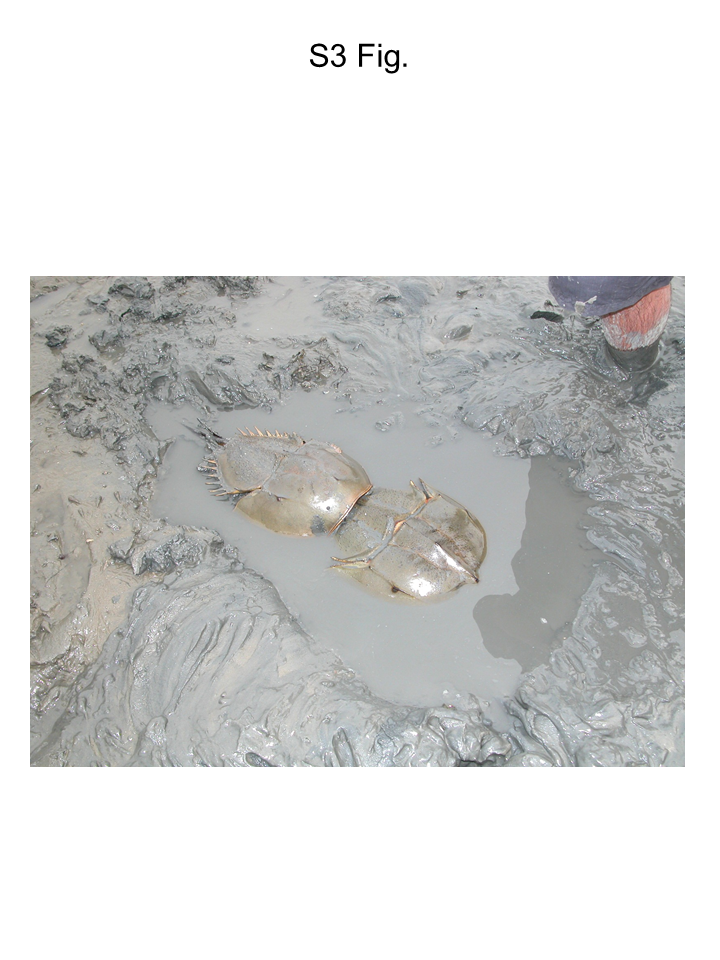

Supplement: S3 Fig — (TIF) [file pone.0147429.s003.tif]
